# Single-Cell Transcriptomics Uncover Key Regulators of Skin Regeneration in Human Long-Term Mechanical Stretch-Mediated Expansion Therapy

**DOI:** 10.3389/fcell.2022.865983

**Published:** 2022-05-30

**Authors:** Yidan Sun, Luwen Xu, Yin Li, Jian Lin, Haizhou Li, Yashan Gao, Xiaolu Huang, Hainan Zhu, Yingfan Zhang, Kunchen Wei, Yali Yang, Baojin Wu, Liang Zhang, Qingfeng Li, Caiyue Liu

**Affiliations:** ^1^ Department of Plastic and Reconstructive Surgery, Shanghai Ninth People’s Hospital, Shanghai Jiao Tong University School of Medicine, Shanghai, China; ^2^ CAS Key Laboratory of Tissue Microenvironment and Tumor, Shanghai Institute of Nutrition and Health, Chinese Academy of Sciences, University of Chinese Academy of Sciences, Shanghai, China; ^3^ Department of Orthopedics, Shanghai Xinhua Hospital, Shanghai Jiao Tong University School of Medicine, Shanghai, China; ^4^ Department of Dermatology, Shanghai Ninth People’s Hospital, Shanghai Jiao Tong University School of Medicine, Shanghai, China; ^5^ Department of Laser Cosmetology, Shanghai Ninth People’s Hospital, Shanghai Jiao Tong University School of Medicine, Shanghai, China; ^6^ Department of Plastic Surgery, Shanghai Huashan Hospital, Fudan University School of Medicine, Shanghai, China; ^7^ Institute for Stem Cell and Regeneration, Chinese Academy of Sciences, Beijing, China

**Keywords:** skin regeneration, mechanical stretch, tissue expansion, single cell RNA sequence, epithelial to mesenchymal transformation, c-fos, AREG–EREG ligands

## Abstract

Tissue expansion is a commonly performed therapy to grow extra skin *in* *vivo* for reconstruction. While mechanical stretch-induced epidermal changes have been extensively studied in rodents and cell culture, little is known about the mechanobiology of the human epidermis *in vivo*. Here, we employed single-cell RNA sequencing to interrogate the changes in the human epidermis during long-term tissue expansion therapy in clinical settings. We also verified the main findings at the protein level by immunofluorescence analysis of independent clinical samples. Our data show that the expanding human skin epidermis maintained a cellular composition and lineage trajectory that are similar to its non-expanding neighbor, suggesting the cellular heterogeneity of long-term expanded samples differs from the early response to the expansion. Also, a decrease in proliferative cells due to the decayed regenerative competency was detected. On the other hand, profound transcriptional changes are detected for epidermal stem cells in the expanding skin versus their non-expanding peers. These include significantly enriched signatures of C-FOS, EMT, and mTOR pathways and upregulation of AREG and SERPINB2 genes. CellChat associated ligand-receptor pairs and signaling pathways were revealed. Together, our data present a single-cell atlas of human epidermal changes in long-term tissue expansion therapy, suggesting that transcriptional change in epidermal stem cells is the major mechanism underlying long-term human skin expansion therapy. We also identified novel therapeutic targets to promote human skin expansion efficiency in the future.

## Introduction

As the outmost barrier of our body, skin has a high demand for renewal and regeneration during physiological growth stimuli and following injury (Biggs et al., 2020). Mechanical stretch-mediated tissue expansion is the epitome of capitalizing on skin’s mechanobiological characterization to repair defects and scar reconstruction in plastic and reconstructive surgery (Zöllner et al., 2013). Ordinary routine expansion includes procedures such as surgically inserting expanders beneath the skin, weekly filling expanders by serial injection of sterile isotonic saline solution over a period, and gradually generating extra skin *in situ* (Huang et al., 2011; Zhu et al., 2018; Topczewska et al., 2019). Thus, we demonstrated that human skin expansion therapy follows a moderate constant volume skin expansion paradigm (MCVSE), in which a moderate stretching force is applied progressively and the skin is maintained in an expanded stage throughout the whole treatment process. It is a very slow process to induce skin regeneration that usually takes at least 8–12 weeks to achieve 3-fold skin growth, but this gradual expansion permits better tissue tolerance. Notably, the clinical expansion is usually capped at about 4-fold while parts of the skin become extremely thin, implicating exhaustion of skin regeneration competency during long-term expansion (LTE) therapy (Li et al., 2016; Huang et al., 2021, 1; Tepole et al., 2012).

Additionally, the rapid skin expansion process (RSE) based on a self-inflating tissue expander is also used in clinical therapy to avoid multiple injections and reduce expansion-related discomfort (Obdeijn et al., 2009). However, once the expansion has begun, there is no possibility to adjust the expansion speed, and difficulties are presented if there are any problems with the overlying skin viability (Lohana et al., 2012). Therefore, choosing between slow or rapid expansion, and small or large inflation volumes, remains controversial, and gold-standard procedures for reconstructive surgery are still under debate (Lee et al., 2018). Recently, a seminal study (Aragona et al., 2020) focusing on the RSE process employed a subcutaneous rapid inflating hydrogel mouse model to explore the mechanism of stretch-mediated skin expansion *in vivo.* Their findings include that stretch induces major changes in epidermal lineage trajectory, including the emergence of a stem cell-like “stretch” cell population that is distinct from regular epidermal basal cells and tips the renewal-differentiation balance to accelerate the production of more stem cells. Also, stretch changed stem cells at the molecular level involved in cell-cell adhesion, actomyosin cytoskeleton, and induced a network of regulatory genes, such as the activation of MEK-ERK–AP1 and YAP-MAL signaling. However, we assume that the mechanistic study of skin therapy based on the RSE model is not enough to closely mimic the changes in human skin in MCVSE clinical settings. Further characterization of the relevance of the main findings from the RSE model to the clinical MCVSE process was warranted. Moreover, exactly how human long-term expansion therapy changes skin phenotypes at the single-cell level in a living organism has not been known, and a better description of LTE would be of absolute importance to the field.

In this study, after a combination of single-cell RNA sequencing (scRNAseq) analysis and two paired long-term expanded skin samples and immunofluorescent (IF) staining of more samples from four individuals from surgical discards, we found that although epithelial lineage identity remains unvaried, numbers of proliferative epithelial cells after long-term expansion decline. Furthermore, they did exhibit prevalent transcriptional changes, particularly in C-FOS, EMT, and mTOR networks, and these were accompanied by skin’s adaption and balance capacity to maintain homeostasis under LTE. Moreover, marked expansion-upregulated genes including AREG and SERPINB2 were found. We also identified that LTE triggered three common pairs of ligand-receptor networks including CD96-NECTIN1, AREG-EGFR, and LAMININ-CD44, and three signaling pathways, including EGF, LAMININ, and NECTIN based on CellChat analysis. Overall, our data present a single-cell atlas of human epidermal changes in long-term tissue expansion therapy, suggesting that transcriptional change in epidermal stem cells is the major mechanism underlying human long-term skin expansion therapy. Our findings also revealed promising therapeutic targets for promoting skin regeneration clinically in the future.

## Methods

### Ethics Statement

The humans expanded and their nearby samples were obtained from discarded plastic surgery specimens in Shanghai’s 9th People’s Hospital (Shanghai, China). Individuals with more than 5 months of the expanded duration were enrolled in the study (*n* = 6). Specifically, samples used for transcriptomic analysis were from two female donors and were collected from different skin areas. They experienced full facial reconstruction with total autologous tissue transplantation and 10-month saline injection. Informed consent from all patients and/or guardians was signed before sample collection in accordance with the Declaration of Helsinki and with approval from the Human Research Ethics Committee of Shanghai Jiao Tong University School of Medicine (Shanghai, China). Detailed patient information is summarized in Table 1.

**TABLE 1:** Clinical information of patients whose samples were used in this study.

Sex	Age (y)	Site	Race	Expanded duration (m)	scRNA-seq analysis	IF staining	Abbreviations
Female	19	Face	Han nationality	10	Yes		F-Exp and F-Nby
Female	23	Neck	Han nationality	10	Yes		N-Exp and N-Nby
Male	18	Head	Han nationality	9		Yes	M18 H9
Female	25	Back	Han nationality	11		Yes	F25 B11
Male	16	Back	Han nationality	13		Yes	M16 B13
Female	17	Neck	Han nationality	14		Yes	F17 N14

### Tissue Dissociation and Cell Isolation

Skin samples were collected and stored in ice-cold phosphate buffer saline (PBS, Sigma-Aldrich) after surgery. For epidermis isolation, subcutaneous tissues were removed from samples and the epidermis was enzymatically dissociated from the dermis with dispase digestion (90% DMEM, 10% Fetal Bovine Serum, 2 mg/ml dispase II, 1% penicillin-streptomycin solution) incubation at 37°C overnight. Epidermal sheets were then manually separated from the dermis and then dissociated into single cells with trypsin-versene (Lonza) incubation at 115 rpm for 10 min at 37°C. The generated single-cell suspensions in 50 ml of 0.04% bovine serum albumin (BSA, Gibco) in PBS were used for ×10 Genomic sequencing.

### Single-Cell RNA Sequencing

The cell suspense dissociated above was loaded into ×10 Chromium controller to generate GEMs with gel beads. The GEMs first reversed transcript to cDNA and then further processed into single-cell 3′ gene expression libraries according to the manufacturers’ instruction manual. In short, the GEMs were reverse transcribed to ss-cDNA first. The single-strand cDNA was purified by beads and amplified by PCR to generate the ds-cDNA. Next, ds-cDNA was fragmented, end-repaired, and further ligated with an adaptor. Lastly, index PCR was performed before sequencing. Sequencing was performed on the Illumina Nova-seq platform.

### Single-Cell Transcriptomic Analysis

Raw FASTQ files were first trimmed by TrimGalore with the parameter “-q 30–phred33–stringency 3–length 20-e 0.1”. Clean FASTQ was processed by the Cell Ranger (v4.0.0) pipeline. After obtaining the UMI matrix, Seurat (v4.0.5) was used for filtering and preprocessing the data. Quality control was performed using the subset function using the threshold of nFeature_RNA larger than 800 and less than 10,000, as well as the percentage of expressed mitochondrial genes less than 20% to filter out low-quality cells and potential doublets. To visualize the data in UMAP and clustering cells, we set the final resolution to 0.5 (testing a range from 0.2–1.0) and dims to 34 (testing a range from 10–50).

### Data Integration

To compare the single-cell RNA-seq data from the adjacent expansion and nearby skin samples, data integration was performed using the CCA algorithm. 2,400 features were selected to find anchors between samples. Further downstream analyses such as dimensionality reduction and clustering were all performed as described.

### Trajectory Analysis

The pseudotime analysis was done by Monocle3 (Cao et al., 2019) and scVelo (Bergen et al., 2020) to reconstruct the epidermal cell developmental trajectory. According to prior knowledge of differentiation from basal cells to spinous cells, the “root” cell is chosen at the very beginning of basal cell in Monocle3. The IFE cells were further divided into three stages according to the pseudotime and defined as “EDC”, “MDC,” and “LDC”. To map the differentiation trajectory directions, scVelo was used to calculate the RNA velocity. The cell filter mentioned above was used to calculate the transcriptional dynamics of splicing kinetics. The standard dynamical modeling workflow was used to obtain the stream plot of velocities.

### Gene Ontology Analysis

GO analysis of DEGs was performed by Metascape (version 3.5, http://metascape.org/) and visualized with the ggplot2 R package (https://github.com/tidyverse/ggplot2) and prism 9. Representative terms of biological processes (BP) selected from the top 20 ranked GO terms or pathways (*p* < 0.01) were displayed.

### Gene Set Enrichment Analysis

The gene set enrichment analysis was performed using the single seqgset R package (https://arc85.github.io/singleseqgset/), which uses a simple underlying statistic (variance inflated Wilcoxon rank-sum testing) to determine the enrichment of gene sets of interest across clusters. The 50 hallmark genesets are downloaded from MSigDB Collections. Other self-defined genesets were listed in Supplementary Table S1.

### Cell-Cell Communications

To infer the intercellular communication network between clusters, the CellChat R package was used to quantitatively measure networks through the law of mass action based on the average expression values of a ligand by one cell group and that of a receptor by another cell group, as well as their cofactors. Significant interactions are identified on the basis of a statistical test that randomly permutes the group labels of cells and then recalculates the interaction probability. We also compared the cell–cell communication probability between the Exp and Nby samples. The DEGs calculated by the *identifyOverExpressedGenes* function were mapped onto inferred cell-cell communications to subset the significantly changed ligand-receptor pairs. Furthermore the upregulated pairs were shown in the bubble plot.

### Immunofluorescence Staining

For IF staining of sections, cryosections were made from frozen tissues embedded in OCT compound (Tissue Tek). The paired tissues were prepared on the same slide to ensure the same staining conditions. Slides were fixed for 10 min in 4% paraformaldehyde and blocked for 1 h in blocking buffer (2.5% normal donkey serum+2.5% normal goat serum+1% BSA+0.3% Triton X-100). Sections were then incubated with primary antibodies at 4°C overnight and with fluorochrome-conjugated secondary antibodies at room temperature for 1 h. Slides were then washed in PBS and mounted with Fluoromount-G mounting media (Invitrogen). Images were taken by Zeiss LSM 880 upright confocal multiphoton microscope. The following antibodies dilutions were used: KRT14 (chicken, Biolegend, 1:500), KRT15(mouse, Santa Cruz, 1:500), KRT10 (rabbit, Abcam, 1:500), KI67 (rabbit, Abcam, 1:500), PCNA(mouse, Servicebio, 1:200), BCL-2 (mouse, Biolegend, 1:200), CD45 (mouse, Biolegend, 1:200), E-CAD (rat, eBioscience, 1:200), Phospho-mTOR (Ser2448) (rabbit, CST, 1:50), FOS (rabbit, Abcam, 1:200), P63 (rabbit, Abcam, 1:200), AREG (rabbit, Abcam, 1:200), SERPINB2 (mouse, Novusbio, 1:200), NECTIN1(rabbit, Thermofisher, 1:100), CD96 (mouse, Santa Cruz, 1:50).

### Immunofluorescence Intensity Measurement

All photographs were taken at the same exposure time. To quantify the intensity of the immunostaining for E-CAD at the adherens junctions, we used the pseudo-color Fire from ImageJ, a well-established method of measuring fluorescence intensity that accounts for differences in the area of the signal (Ellis et al., 2019; Aragona et al., 2020), and the integrated density signal was shown in the figures.

### Statistical Analysis

For quantification of the thickness of the epidermis and papillary dermis (PD), we used the total area divided by the length of the basal membrane to get the average thickness. For quantification of the staining intensity, the expanded and their nearby counterparts were cut and placed on the same slice. They were photographed with the same exposure duration and light intensity on the same microscope mentioned before. All the results were analyzed with ImageJ software (National Institutes of Health, Bethesda, MD). Comparisons across multiple groups were made using a two-way ANOVA with the Sidak’s multiple comparisons post hoc test. Differences were regarded as significant at *p* < 0.05. The quantitative data shown is expressed as mean ± standard error of the mean (SEM, represented as error bars). Graphpad Prism 9 software was used to assess statistical significance. The statistical significance level was set at *p* < 0.05.

## Results

### scRNA-Seq Analysis Revealed Similar Cell Type Compositions Under Long-Term Expansion at Single-Cell Resolution

**FIGURE 1:**
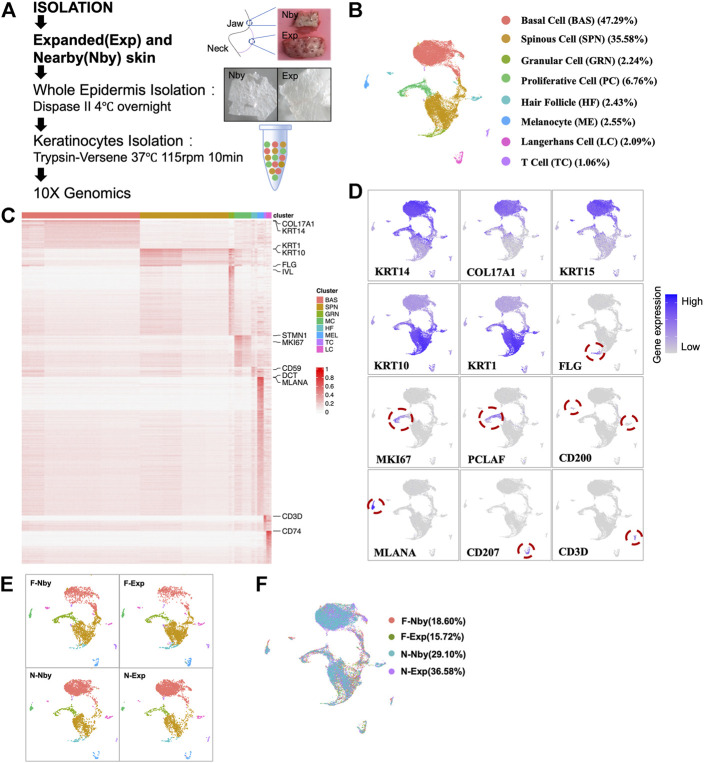
ScRNA-seq reveals similar cell type composition in epidermal keratinocytes after long-term stretch. **(A)** Overview of the experimental workflow. Paradigm of single-cell isolation and sequencing strategy (right). White field images of the expanded and nearby skin of humans (left). **(B)** Uniform manifold approximation and projection (UMAP) cluster of epidermal cells (*n* = 22223) from exp and its nby skin. Major cell types are classified using marker genes and colors corresponding to cell identity. **(C)** Heatmap of differentially expressed genes. Selected genes for each cluster are shown on the right. **(D)** UMAP plots show the representative marker genes of each cell type in human skin. The color key from gray to purple indicates low to high gene expression levels. **(E)** UMAP plot presents 3,000 cells and the same cell types in each of the four samples. Cells are colored by sample, and the percentage of each sample is annotated to the right. **(F)** UMAP plot showing similar patterns of cell clustering for each sample.

To date, seminal studies have focused on the instantaneous responses of mechanical stretch (Le et al., 2016; Aragona et al., 2020; Nava et al., 2020). Although previous research verified that skin regenerative regulation induced by mechanical stretch was time-correlated and 5-month-expansion was defined as LTE (Wang et al., 2021, 2), a detailed analysis of the LTE phenotypes of humans under clinical settings is still needed. To determine the changes in human skin epidermis during long-term (>5 months) tissue expansion therapy, we collected samples of human facial (F) and neck (N) LTE skin, respectively. Each contained paired samples from the expansion (Exp) and nearby non-expansion area (Nby) (Table 1). The epidermis was isolated by dispase digestion from these samples and subjected to 10x Genomics single-cell RNA-sequencing (scRNAseq) (Figure 1A). After stringent cell filtration, 22,223 cells were retained for subsequent analyses. We visualized human epidermal cell populations using uniform manifold approximation and projection (UMAP) (Figure 1B) and identified 8 major cell clusters based on the expression of classic skin lineage markers, including basal cell (BAS, *KRT14+*, *KRT15+*, *COL17A1+*), spinous cell (SPN, *KRT10+*, *KRT1+*), granular cell (GRN, *FLG+*, *IVL+*), proliferative cell (PC, *KI67+*), hair follicle (HF, *CD59* ^*+*^, *CD200+*), melanocyte (ME, *DCT+, MLANA+*), Langerhans cell (LC, *CD207+*), and T cell (TC, *CD3D+*) (Figures 1C,D). Besides melanocytes and immune cells, the epidermal compartment contains a clear basal cell group (BAS) that is *KRT14+KRT15+KRT10-*, a proliferative cell group (PC), several differentiated cell groups, and some intermediate cells between BAS and differentiated cells. Despite the lower number of cells, the individual analysis of each sample generated a similar distribution of clusters and identified the same major cell types, which means there is no distinct “stretch” cell group found in the Exp samples (Figures 1E,F). Overall, no obvious changes in the cellular compositions were detected in Exp samples versus their Nby counterparts.

### Epidermal Cells Showed No Overt Signs of Lineage Trajectory Alternation After Long-Term Expansion

**FIGURE 2:**
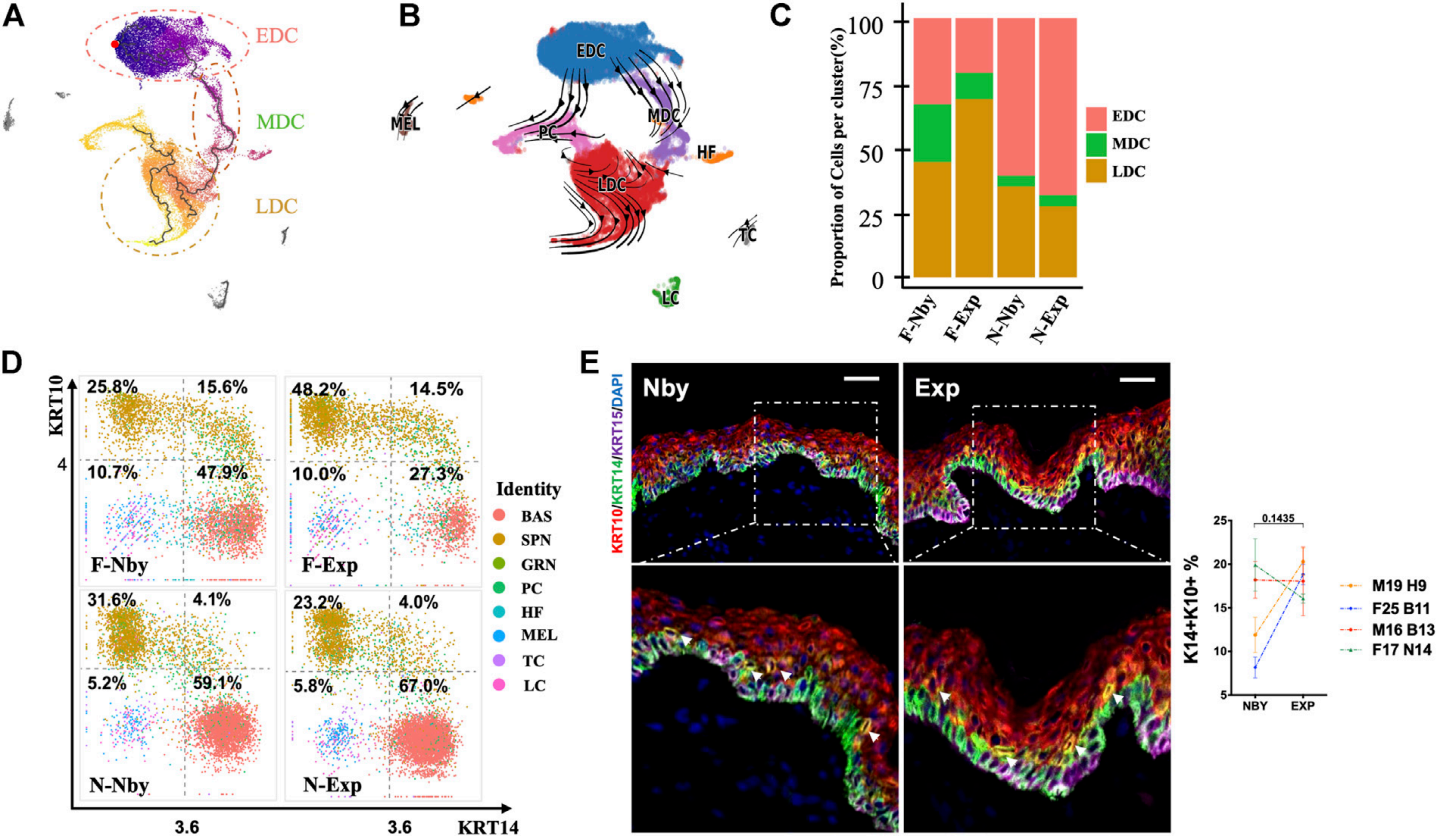
Epidermis maintained a similar lineage trajectory to its counterparts after LTE. **(A)** Monocle3 pseudotemporal visualization of all the epidermal cell types along differentiation trajectories from dark purple to light yellow. The root cell was indicated in red, and the edges are shown as black line segments. Clusters were defined from early to late according to the rank of pseudotemporal cell ordering. **(B)** Velocities derived from the dynamical model for epidermal differentiated trajectory are visualized as streamlines in a UMAP-based embedding. Cells are color-coded by clusters. **(C)** The fraction of redefined clusters in each sample. **(D)** Two-dimensional scatter plots of KRT14 and KRT10 expression in all cell types. Note that there is no increasing fraction of KRT14^high^ and KRT10^high^ cells located in the right upper quadrant (RUQ) after long-term expansion. **(E)** Representative IF images of the nearby and expanded skin. KRT14 and KRT10 co-expressed cells (yellow with arrows) and the quantification of their fraction in epidermal cells.

Stretch-mediated skin expansion is observed as a temporary increase in stem-cell division and eventually fuels the stem-cell differentiation as extra skin is generated. Furthermore, there exists a “stem cell-like stretch cluster” as a fast-responding population of the basal stem cell (Aragona et al., 2020). To analyze the epidermal differentiation dynamics and lineage trajectory in long-term expanded epidermis, we performed pseudotime analysis using Monocle3, in which an epidermal lineage trajectory that starts with BAS cells (Early Differentiated Cluster, EDC), passes through the intermediate cells (Middle Differentiated Cluster, MDC) and ends in differentiated cells (Late Differentiated Cluster, LDC) (Figure 2A). This prediction was coincident with the classic model for epidermal differentiation by previous scRNA-seq studies of human skin (Cheng et al., 2018). Meanwhile, we performed RNA velocity analysis to predict the potential directionality and transitional state of the epidermal cells (Figure 2B). We can see a branch trajectory with two major branches in EDC, which develop into both PC and MDC. A small fraction of the upper LDC displayed the velocity vectors pointing toward PC as the terminal, indicating that both EDC and upper LDC are capable of proliferation. Furthermore, the trajectory of LDC involves the development of both committed cells and terminally differentiated cells. These findings were consistent with the classic model of epithelial self-renewal and differentiated trajectory (Fuchs, 2008) but highlighted the importance of PC in skin homeostasis under stretch conditions. In contrast to the nby epidermis, cells in expanded groups identified by the differentiated stage showed inconsistent changes in numbers (Figure 2C). Thus, we concluded that long-term expanded human skin epidermis maintained a normal lineage trajectory. Besides, in adult primate skin, it takes ∼4 weeks for a committed epidermal cell to exit the basal layer and be sloughed from the skin surface (Gonzales and Fuchs, 2017). It has “committed progenitors” belonging to basal daughters but expressing dual-differentiated markers. Researchers referred to the dually positive KRT14^+^KRT10^+^ cells as differentiated-poised state cells with asymmetric fate outcomes (Asare et al., 2017). The short-term expansion is always accompanied by the thickening of the epidermis, suggesting the activation of the differentiated process of the basal stem cells (Zöllner et al., 2013; Huang et al., 2021, 1). Considering KRT14^+^KRT10^+^ cells are the committed cells poised for differentiation, we aim to gain insight into how the differentiation tendency of the epidermal is controlled under LTE at the single-cell level. So, we observed the committed populations based on the transcriptional expression of *KRT14* and *KRT10*. Relative enrichment of committed cells (*Krt14* ^*+*^ *Krt10* ^*+*^) was not observed in Exp vs. Nby comparisons for both sample pairs (Figure 2D). Further double staining of KRT14 and KRT10 also showed the comparable abundance of *KRT14+KRT10+* cells in Exp vs. Nby skin (Figure 2E). Together, these data indicate that the epidermal lineage trajectory is not significantly altered and maintains homeostasis behavior in LTE human skin under clinical conditions.

### Long-Term Expansion Induced Decayed Proliferative Activities of the Epidermis

**FIGURE 3:**
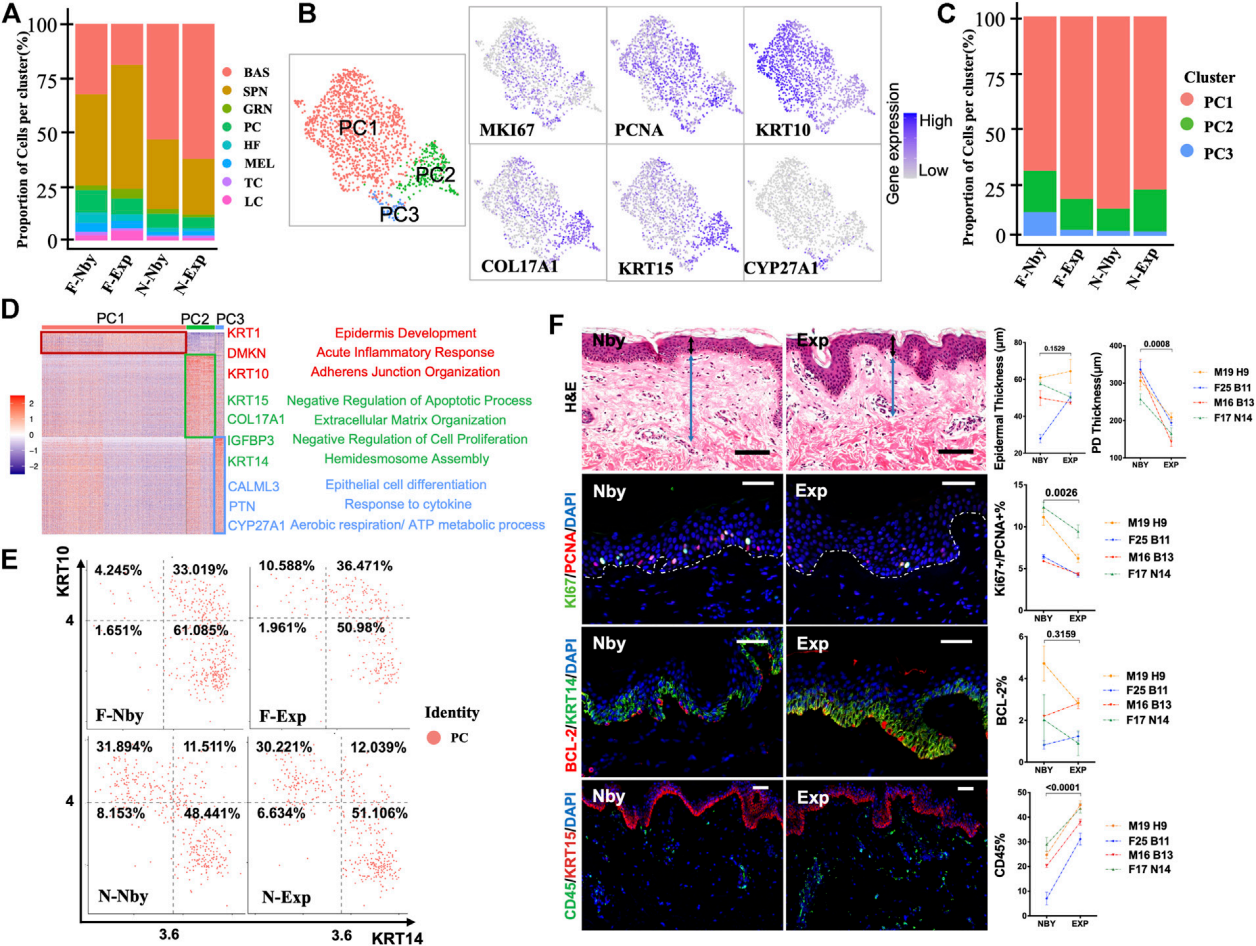
LTE-induced decayed proliferative activities of the epidermis. **(A)** The proportion of cells for each of the eight cell types in each sample. Note the decreased fraction of the PC cell. **(B)** UMAP plots showing the subpopulations of proliferative cells (PC) with the expression of representative genes are shown to the right. **(C)** The proportion of the proliferative subpopulations in each sample. Note the inconsistent changes in the proportion between the samples. **(D)** Heatmap showing the scaled expression levels of genes highly expressed specifically in PC1, PC2, and PC3. The color key from blue to red indicates low to high gene expression levels. Cell-type-specific representative genes and GO terms are listed to the right. **(E)** Two-dimensional scatter plots of KRT14 and KRT10 expression in PC. **(F)** Representative images of the nby and exp skin. The sections belong to four individuals undergoing long-term tissue expansion. Two-way ANOVA was applied in statistical data analysis and *p* < 0.05 was considered statistically significant. Scale bars = 50 μM. All parameters were the same in all subsequent figures unless otherwise mentioned. First row: H&E-stained skin nearby, expansion skin, and quantification of the thickness of the epidermis, and papillary dermis (PD). A black bidirectional arrowhead refers to the thickness of the epidermis and a blue bidirectional arrowhead refers to the thickness of PD. Second row: IF staining images of proliferative KI67^+^ and PCNA^+^ cells and quantification of their percentage in epidermal cells. Third row: IF staining images of apoptotic BCL-2^+^ cells and quantification of their percentage in epidermis cells. Last row: The inflammatory CD45^+^ cells and the quantification of their fraction in dermal cells.

In contrast to the nby groups, the fraction of epidermal cells in exp groups identified by clusters showed comparable changes in numbers but displayed strikingly consistent reductions in the proportion of PC clusters (%) (F-Nby vs. F-Exp: 10.26 vs. 7.39 and N-Nby vs. N-Exp: 6.45 vs. 5.01) (Figure 3A), which was reminiscent of cell growth arrest after LTE (Wang et al., 2021). Furthermore, not all samples had a reduction in the number of basal stem cells after LTE, which was different from our expectations (Figure 3A). Proliferating epidermal cells (PC) were further subdivided into three subpopulations (PC1–3) based on the previously defined signature genes(Zou et al., 2021) (Figures 3B,C). Endowed with the largest number of cells, PC1 was defined by the high expression of suprabasal keratins KRT1 and KRT10, which are the earliest markers for keratinocytes’ differentiation, indicating that renewal spinous cells were the most sensitive subpopulation to take on stretch-mediated regenerative state for long-term stretch. Also, the PC1 located in the suprabasal layer possessed the ability to respond to the acute inflammatory response reviewed by the GO enrichment analysis (Figure 3D). Combined with the highest expression of COL17A1 and KRT15, we inferred that PC2 was most likely the basal cells. We noticed that PC2 was enriched in upregulated GO in terms of negative regulation of cell proliferation and apoptosis. These upregulated GO terms were in agreement with the decaying tendency of cell proliferation in long-term expanded epidermis (Figure 3D). PC3 occupied the least proportion of proliferative cells with the highest expression of CALML3, PTN, and CYP27A1 (Figures 3B,D). According to the previous finding, it was regarded as the self-renewing state of the most quiescent basal cell and the inflammation-responsive subpopulation (Zou et al., 2021). Our enrichment confirmed that PC3 participated in epithelial differentiation, response to the cytokine, and energy production (aerobic respiration and the ATP metabolic process). According to previous findings, the daughter cells of proliferative cells discriminate in favor of renewal (KRT14 + KRT10-−) rather than commitment to differentiation (KRT14 + KRT10+) (Aragona et al., 2020). Therefore, further analysis of the PC cell group showed that it contains three subgroups that may represent proliferating cells biased for renewal (KRT14^hi^KRT10^low^), differentiation (KRT10^hi^KRT14^low^), and committed cells (KRT10^hi^KRT14^hi^) (Figure 3E). However, no consistent changes in the relative abundance of the renewal subgroups but a slight increase in the fraction of committed cells was observed in Exp vs. Nby comparisons for both sample pairs after LTE, which is different from the RSE findings (Figure 3E). To verify these findings, we analyzed skin sections from additional independent LTE skin samples. H&E staining and immunofluorescence staining (IF) showed reduced thickness of the papillary dermis (PD) and increased CD45^+^ immune cells in Exp vs. Nby skin (Figure 3F). These are consistent with the known properties of stretching skin (Zöllner et al., 2013; Aragona et al., 2020). However, the epidermal thickness after LTE was not constantly increased but reached a plateau or even decreased. To testify to the fraction of the PC subpopulation, staining of Ki67 and PCNA showed reduced expression of the proliferation marker in Exp vs. Nby skins (Figure 3F). Also, no comparable change in apoptosis (BCL-2^+^) was detected, excluding the possibility of stretch-induced damage (Figure 3F). Overall, our finding provides an in-depth understanding of skin’s adaption to long-term stretch-mediated regeneration and hints at the risk factors for complications of long-term expansion therapy.

### Identification of Transcriptomic Patterns Involved in the Regenerative Process of Long-Term Expansion

**FIGURE 4:**
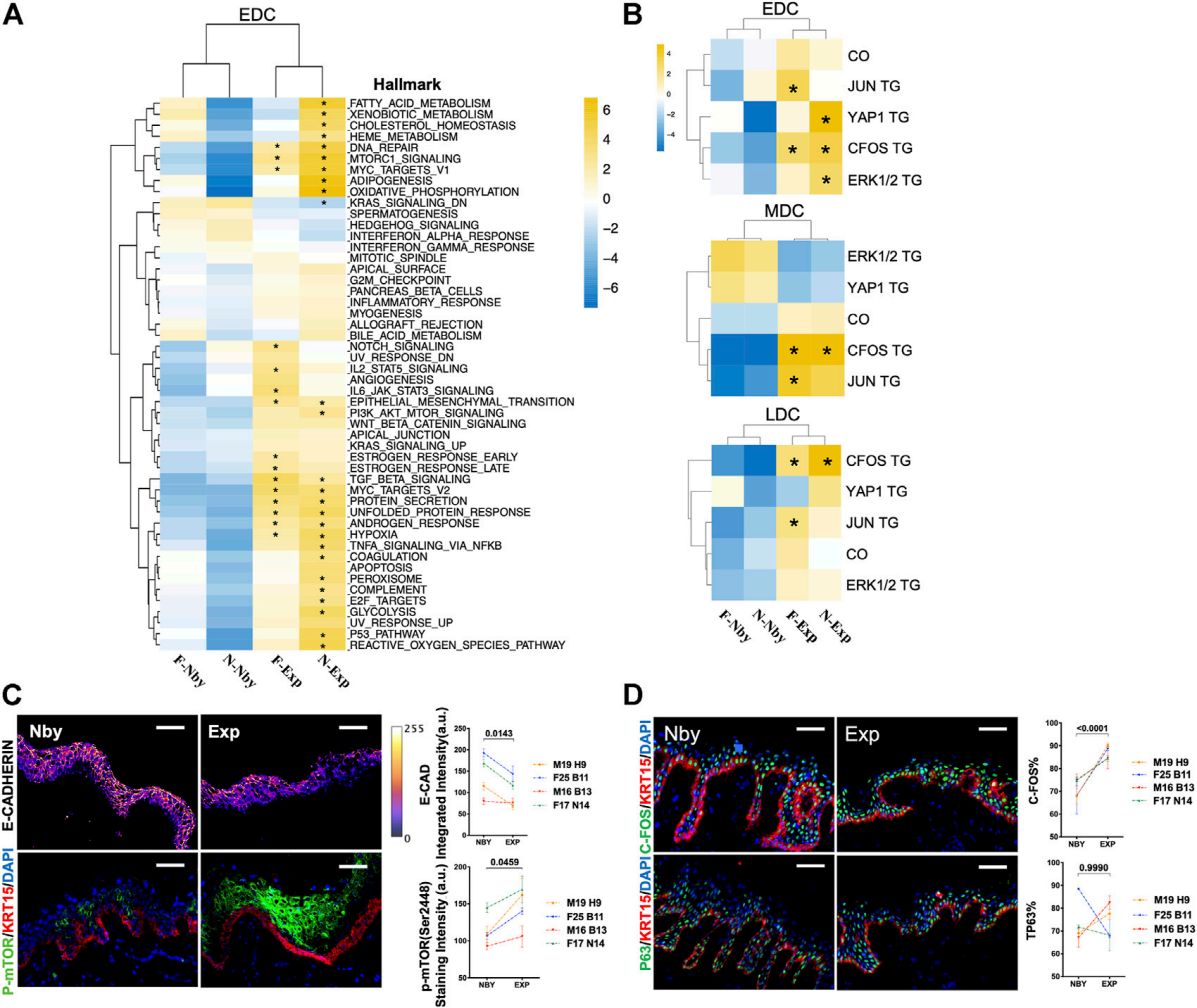
Changes in the transcriptional profiles of the EDC subpopulation during LTE. **(A)** Heatmap showing the GSEA results of the MSigDB hallmark terms in EDC in four samples. *p* < 0.05 is considered statistically significant, same in all following figures unless otherwise mentioned. **(B)** Heatmap showing the GSEA results in of the previously published TGs of the transcriptional factors JUN, C-FOS, YAP1, and ERK1/2 cascade downstream signatures and GO terms of cytoskeleton organization. **(C)** Representative images of the E-CAD and p-mTOR staining in nby and exp samples from four other individuals and their quantification. The E-CAD IF staining intensity was shown by the pseudo-color integrated density signal. The color key (right) from purple to yellow indicates low to high integrated density signal levels. A.U, arbitrary units. **(D)** Representative IF images of the C-FOS and P63 staining in nby and exp skin and their quantification.

By Gene Set Enrichment Analysis (GSEA) analysis for significant enriched Msigdb hallmarks (Wu and Smyth, 2012) in the EDC group of each sample relative to other samples, we found that F-Exp and N-Exp EDCs are transcriptionally similar to each other but are distinct from F-Nby and N-Nby EDCs (Figure 4A), suggesting a specific transcriptional signature for epidermal stem cells under stretching. Among these, Exp EDC was significantly enriched for epithelial-mesenchymal transition (EMT), mTORC1 signaling, DNA repair, hypoxia, MYC targets, protein secretion and TGF-β signaling hallmarks, which partly are reported to be involved in epidermal stretching response (Liang et al., 2014; Zhou et al., 2020). Whereas the inflammatory infiltration during the long-term stretch was not enriched significantly, this was different from the highly inflammatory response of the short-term stretch (Aragona et al., 2020; Ledwon et al., 2020). The previous mouse study identified *AP-1, YAP, ERK* signaling, and cytoskeleton organization (CO) pathways as key drivers of stretching-induced skin growth *in vivo* (Aragona et al., 2020). To verify, we performed additional GSEA analysis using reported target genes (TG) of *C-FOS* (GSE10218, Durchdewald et al., 2008), *JUN* (GSE119762, Gago-Lopez et al., 2019), *YAP1* (GSE137531, Yuan et al., 2020), *ERK1/2* (GSE15417, Dumesic et al., 2009) and the GO ontology term of CO (7010, Supplementary Table S1). The results showed visible enrichment of most of these genesets in Exp vs. Nby EDCs (Figure 4B). Among these, C-FOS TGs are most consistently enriched in both Exp samples and also in MDC and LDC cell groups (Figure 4B). To verify these findings, we again performed IF analysis on independent clinical LTE samples. The data confirmed significant reduced E-CAD + expression (a classic EMT marker) (Zeisberg and Neilson, 2009), enhanced phospho-mTOR expression (activated mTORC1 signal marker) (Obara et al., 2015) (Figure 4C), and elevated C-FOS expression in Exp vs. Nby comparisons, while epidermal progenitor marker P63 (Soares and Zhou, 2018) remained unchanged (Figure 4D). Taken together, transcriptomic patterns are indeed altered jointly after LTE despite genetic specificities between the two samples used for scRNAseq.

### Gene Expression Changes in Human Epidermal EDC After Long-Term Expansion

**FIGURE 5:**
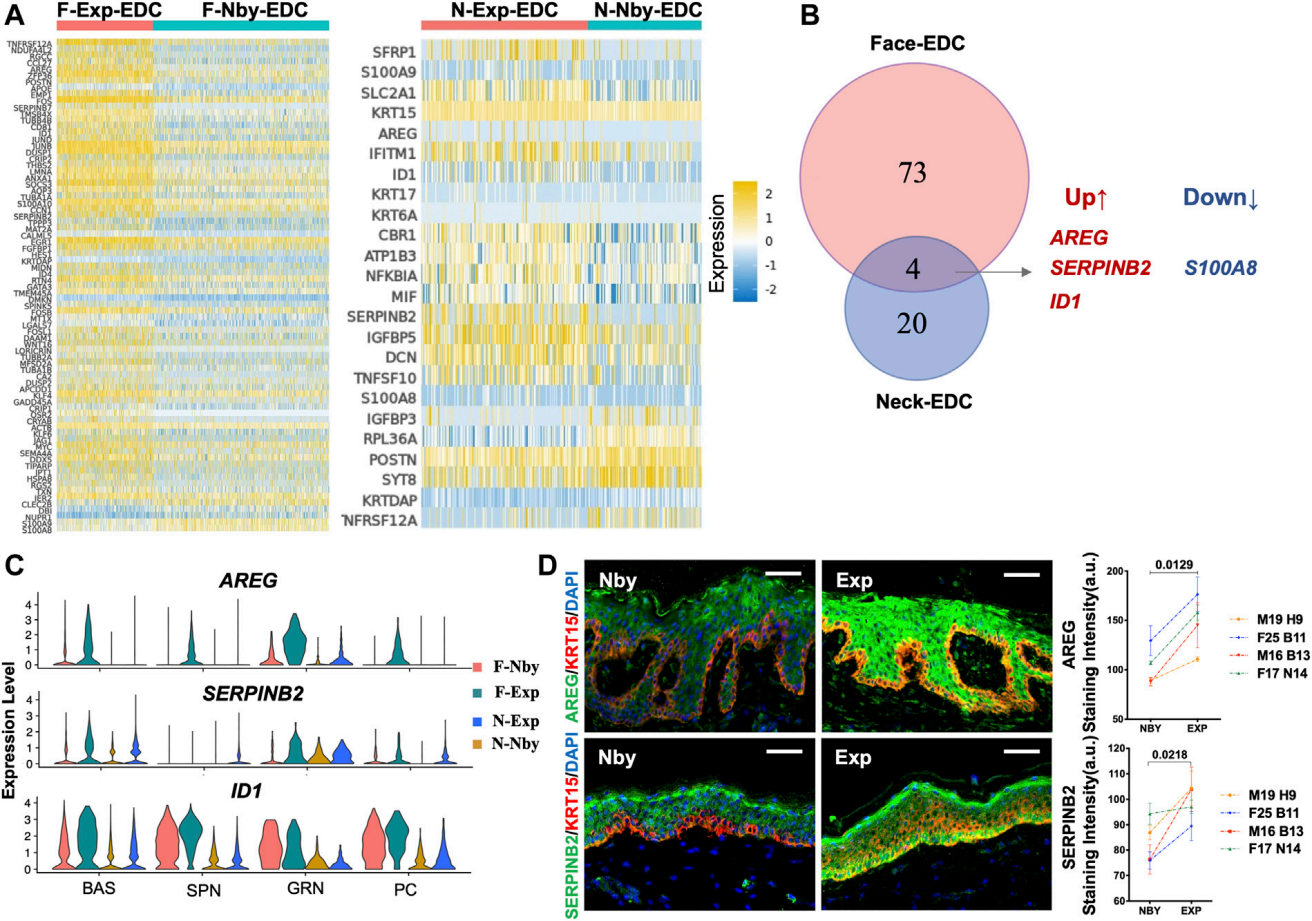
Identification of the differentially expressed genes (DEGs) in the EDC subpopulation during LTE. **(A)** Heatmaps showing the DEGs identified in EDC between the exp and nby groups. The color key from blue to yellow indicates low to high gene expression levels. Representative genes are listed to the left (|FC| ≥ 0.5). **(B)** Venn diagram showing the overlap between DEGs in Face-EDC vs. Neck-EDC (|FC| ≥ 0.5). **(C)** Violin plots showing the expression level and distribution of the selected DEGs in subpopulations (BAS, SPN, GRN) and proliferative cells (PC) in four samples. **(D)** Representative IF images of the AREG and SERPINB2 staining in nearby and expanded skin from four other individuals and their quantification.

To gain insights into the mechanisms of skin regeneration after LTE, we next examined the Exp vs. Nby changes of individual genes in the EDCs. Since the facial and cervical specimens were from different individuals and anatomic sites, we expected they would endow a high degree of heterogeneity between the samples. Differentially regulated genes (DEG) were considered as having an absolute fold change (|FC|) ≥ 0.5 (Figure 5A). As expected, the facial sample contained more DEGs than the cervical samples. Overlap between the DEGs, including only three upregulated genes and one downregulated gene, was observed in both sample pairs (Figure 5B). Accordingly, the violin plot shows the transcriptomic distribution of the upregulated genes (Figure 5C). IF analysis confirmed significant upregulation of Amphiregulin (AREG) and SERPINB2 in Exp vs. Nby comparison at the protein level (Figure 5D). Notably, *S100A8*, the downregulated gene, was the damage-associated molecular in skin disorders and will be secreted under inflammatory microenvironment (Defrêne et al., 2021). Its downregulation suggested the degree of inflammatory response was attenuated over time. Generally, whether they are novel regulators of clinical skin expansion would be an interesting topic for future analysis.

### Identification of Major Signaling Changes in Long-Term Expanded Human Epidermis

**FIGURE 6:**
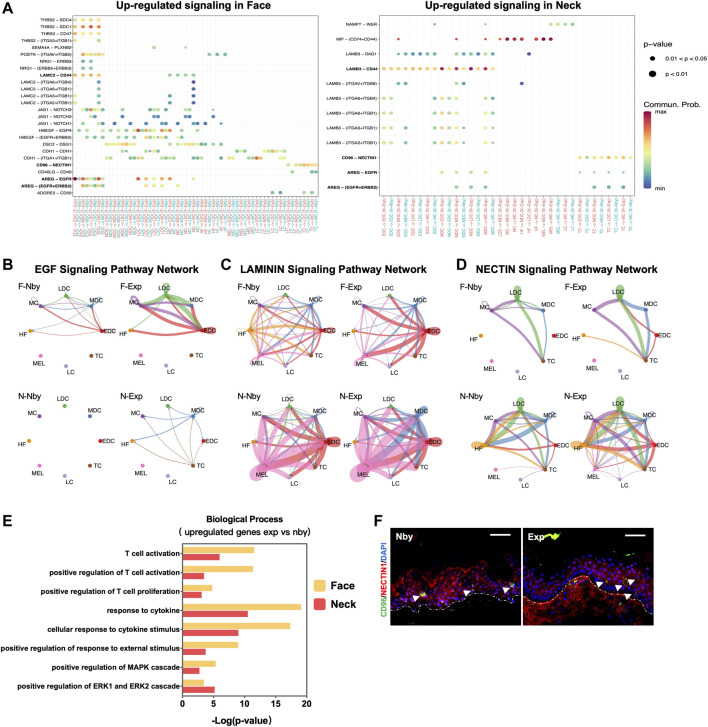
Intercellular ligand–receptor prediction. **(A)** Visualization and comparison of the significant upregulated ligand-receptor pairs in the exp vs. nby groups. Dot color reflects communication probabilities, and dot size represents computed *p*-values. Empty space means the communication probability is zero. *p*-values are computed from a one-sided permutation test. **(B–D)** The inferred EGF, LAMIN, and NECTIN signaling networks. Different cell populations are color-coded. Circle size is proportional to the number of cells in each cell group, and edge width represents the communication probability. **(E)** GO biological process analysis of T cells. **(F)** Representative IF images of the NECTIN1 and CD96 staining in nby and exp skin. Arrows denote CD96 positive cells, and the dotted lines denote the basement membrane between the epidermis and dermis.

Signaling crosstalk via ligands and receptors is critical in tissue development and cellular decisions (Bhattacharjee et al., 2019). To clarify the underlying intercellular communications that drive skin regeneration for long-term expansion therapy, we analyzed intercellular communication networks among the 8 cell groups from scRNA-seq data using CellChat (Jin et al., 2021). It identified ligand-receptor pairs AREG-EGFR, CD96-NECTIN, and LAMIN-CD44 as the most upregulated significant signaling in exp versus nby counterparts, suggesting that these pathways are essential for stretch and likely contribute to the sustainability of long-term skin regeneration (Figure 6A). The downregulated ligand-receptor pairs are shown in Supplementary Figure S1A. Cellchat revealed that the EGF pathway exhibited abundant signaling interactions. But EGF ligands in F-Exp are dominantly secreted by EDC, LDC, and MC, whereas in N-Exp they are secreted by MDC and TC (Figure 6B). LAMB3 and LAMC2 are the ligands of the laminin pathway that regulate the attachment of the basal membrane (Yap et al., 2019). The laminin-CD44 interaction contributes to the increased migration during wound healing (Michopoulou et al., 2020). Therefore, CellChat prediction suggests that LAMININN signaling in EDC and MDC cells plays a central role in directed migration after mechanical stretch (Figure 6C). CD96-NECTIN has been described as influencing the adhesive and migrative function of T cells and negatively controlling their cytokine responses (Seth et al., 2007; Chan et al., 2014, 96). Therefore, we predicted that epidermis-resident T cells may be activated and interact with the epidermal cells during LTE (Figure 6D). To further elucidate the role of epidermis-resident T cells after LTE, we performed GO biological process enrichment analysis using the upregulated DEGs of T cells (log 2 (FC) > 0.3). The common terms of the two samples were listed in Figure 6E, including positive regulation of T cell activation and proliferation, response to inflammatory and immune responses, and positive regulation of MAPK and ERK cascade. The commonly upregulated genes include FOS and JUND, indicating the inflammatory regulation they may participate in LTE (Supplementary Table S2). We further performed IF staining to verify the bioinformatic analysis on cellular communication. As shown in Figure 6F, CD96-NECTIN1 ligand-receptor pairs from the bioinformatic analyses were verified experimentally. Overall, we predicted the biologically meaningful intercellular communications of human LTE from scRNA-seq data.

## Discussion

Our study was designed as a paired study with control samples deriving from preferably comparable skin segments of the same subject. The sample size was adequate to yield a convincing conclusion, although the different anatomic sites exhibited distinct features. Our results not only highlighted the similarities across the spectrum of human LTE but also revealed consistently specific markers, including known signaling pathways and some new expansion-related genes. Functional studies are warranted to follow up on some of the unique molecular pathways identified in this work. Also, single-cell ATAC seq to unravel the chromatin landscape of basal stem cells requires further investigation in future analysis. It will also contribute to an enhanced understanding of the mechanisms of the LTE and the strategies to promote skin regeneration clinically.

Of note, Aragona et al. (2020) in Nature observed that increased stem-cell renewal fuels stem-cell differentiation and stretches the renewal-differentiation balance in favor of producing more stem cells. In our study, we investigated the renewal-differentiation balance of the proliferative cell based on the KRT14 and KRT10 expressions. Opposite to their findings, the renewal of proliferative cells did not show signs of increasing but the committed cells poised for differentiation did after LTE. Also, the thickening of the epidermis after the expansion is often accompanied by the activation of the differentiated process of the basal stem cell. However, we did not observe the increased fraction of the spinous cells or the enrichment of the KRT14^+^KRT10^+^ committed cells. We also expected the stem cell-like stretch cluster to be the fast-responding cluster once the stretch was applied. So, we performed Monocle3 and RNA velocity to predict whether a differentiation trajectory from basal stem cell to this “stretch” cluster exists in our LTE samples or not. However, our results elucidate that the skin composition remains in homeostasis after LTE. Overall, there are fundamental differences between RSE models and clinical MCVSE processes that need further investigation.

For more than 60 years (NEUMANN, 1957), physicians have taken advantage of the particular response and effective interventions of stretch-induced skin expansion. After years of stagnation, it is exciting to develop targets to promote regenerative competency and diminish the complications of LTE. Our finding showed that EMT was the key regulator to promoting regeneration and maintaining stemness in basal keratinocytes during LTE. It should be noted that the hypoxia (Thiery et al., 2009), DNA repair (Weyemi et al., 2016), TGF-β(Kahata et al., 2018), mTORC1 (Gulhati et al., 2011), MYC signal (Cho et al., 2010) were the upstream inducer that favors the activation of EMT process. All activities converge on the induction of the E-cadherin repressor, which has been verified at the protein level. Plus, the *AP-1* transcription factor comprises members of the *FOS* family (mainly *C-FOS*) and the *JUN* family (mainly *C-JUN* and *JUNB*) (Eferl and Wagner, 2003). *c-Fos* was reported to participate in the skin hyperplasia mediated by inflammation, suggesting that inflammation accompanied by LTE may trigger c-fos activation throughout this process (Briso et al., 2013). Additionally, the epidermal growth factor (EGF)-like molecular AREG plays a central role in orchestrating host protection (Zaiss et al., 2015) and epithelial regeneration (Lu et al., 2021). Accordingly, AREG has a huge potential to exert both anti-inflammatory and pro-regenerative effects and minimize the risk of complications during LTE. SerpinB2 maintains the barrier function of the stratum corneum (Schroder et al., 2016), suggesting its positive role in maintaining the integrity of the skin barrier.

It is noteworthy that tissue expansion results in the dermis thinned out (Agrawal and Agrawal, 2012), angiogenesis occurs (Zöllner et al., 2013), altered epithelial-mesenchymal interactions (Zhou et al., 2020, 1), chronic inflammation and fibroblast-myofibroblast differentiation (Kollmannsberger et al., 2018). The latter feature (differentiation of fibroblasts into myofibroblasts) is central to the excessive production of collagen-rich ECM (Tomasek et al., 2002; Tai et al., 2021) and dermal fibrosis (Gao et al., 2019; He et al., 2021) under mechanical stretch. A further study verified that the papillary dermis exerts crucial functions in the favorable prognosis of skin regeneration under stretch (Tan et al., 2022). It is also acknowledged that tissue-resident stem cells are critically important for the development and regeneration of the skin (Gonzales and Fuchs, 2017). Whereas stem cells derived from dermis including neural crest stem cells, MSC-like stem cells, and hematopoietic cells have a lower capacity to participate in the process of skin regeneration compared to epidermal stem cells and hair follicle stem cells (Díaz-García et al., 2021). What’s more, the dermis interdigitates with the epidermis so that both the epithelium and mesenchyme release signal factors that regulate cellular behavior in a reciprocal manner (Russo et al., 2020). However, the presence of keratinocytes tips the balance of skin regeneration by directing ECM remodeling of fibroblasts (Ghaffari et al., 2009) and reconstructing the epithelial appendages (Wu et al., 2014). Thus, we assume that the epidermis plays a more vital role in skin regeneration compared to the dermis and have focused on the phenotypes of the epidermal stem cells in tissue expansion therapy.

However, the selection of the anatomical donor sites for skin expansion can vary considerably depending on different preoperative diagnoses and various operative procedures (Huang et al., 2011; Azzi et al., 2020). Because of the distinct transcriptional programming varied by the anatomical sites (Cheng et al., 2018), it is relatively challenging to exploit a common strategy to enhance the regenerative competency for long-term expansion therapy. Additionally, the expansion treatment process ends when the surface area of the expanded skin equals the sum of the surface area of the donor site plus the area of the adjacent soft-tissue defect, which means the endpoint of treatment meets the needs of surgical therapy rather than ends of the skin’s expanded state. The study on the LTE samples under serial stretch did not reflect the early response of the expansion because any harvesting tissues during the expansion treatment process in the early or in the middle period were not able to meet ethical requirements and affect the patients’ interests, including treatment safety and effects.

## Data Availability

The datasets presented in this study can be found in online repositories. The name of the repository and accession number can be found below: NCBI; PRJNA797897.

## Supplementary Material

Supplementary Figure S1
**(A)** Visualization and comparison of the significant upregulated ligand-receptor pairs in the exp vs. nby groups. Dot color reflects communication probabilities, and dot size represents computed *p*-values. Empty space means the communication probability is zero. *p*-values are computed from a one-sided permutation test.

Supplementary Table S1 List of genes used for GSEA analysis.

Supplementary Table S2 List of jointly upregulated genes of T cells used for GO biological process analysis.
